# Factors associated with colostrum feeding practice among mothers who attended child immunization clinic in Dire Dawa City, Eastern Ethiopia

**DOI:** 10.11604/pamj.2024.48.115.35931

**Published:** 2024-07-18

**Authors:** Natnael Dechasa Gemeda, Firehiwot Mengistu, Fentahun Alemnew Chekole, Wondu Feyisa Balcha, Kebede Feyisa Adugna, Getachew Andualem Belete

**Affiliations:** 1 Department of Midwifery, College of Medicine and Health Sciences, Dire Dawa University, P.O. Box: 1362, Dire Dawa City, Ethiopia,; 2 School of Chemical and Food Engineering, Department of Applied Human Nutrition, Bahir Dar University, P.O. Box: 079, Bahir Dar City, Ethiopia,; 3 Department of Midwifery, College of Medicine and Health Sciences, Bahir Dar University, P.O. Box: 079, Bahir Dar City, Ethiopia,; 4 Department of Pharmacy, College of Medicine and Health Sciences, Bahir Dar University, P.O. Box: 079, Bahir Dar City, Ethiopia,; 5 Bahir Dar Health Sciences College, P.O. Box: 079, Bahir Dar City, Ethiopia

**Keywords:** Bahir Dar University, colostrum, Dire Dawa, knowledge, practice

## Abstract

**Introduction:**

colostrum is yellow to orange-colored milk produced during the first few days of lactation and it is rich in nutrients and antibodies that have a greater role in the health of the newborn baby. Despite the World Health Organization's recommendation that every newborn baby has to be fed breast milk colostrum within one hour of delivery, poor colostrum feeding practice is still a common problem in Ethiopia. Therefore, this study aimed to assess colostrum feeding practice and associated factors among mothers who attended child immunization clinic at the public health facilities of Dire Dawa City, Eastern Ethiopia, 2021.

**Methods:**

a health facilities-based cross-sectional study was employed from February 1/2021 to, March 2/2021 in the public health facilities of Dire Dawa City among 292 mothers. The data were collected by systematic random sampling technique, entered into Epi data 4.2, and analyzed using the Statistical Package of Social Sciences 25.0 version. Bivariate and multivariable logistic regression analysis was employed to estimate the crude and adjusted odds ratio with a confidence interval of 95% and a P-value of less than 0.05, considered statistically significant. Frequency tables and descriptive summaries were used to describe the study variables.

**Results:**

in this study 68.8% [95% CI: 63.4-74.3] of the mothers had good colostrum feeding practices. In multivariable analysis living in urban [AOR=2.52, 95% CI=1.08-5.88], secondary and above education [AOR=2.79, 95% CI=1.12-6.98], having antenatal care visit [AOR=3.12, 95% CI=1.12-9.21], counselled on timely initiation of breastfeeding [AOR=2.59, 95%, CI=1.02-6.59], counselled on colostrum feeding [AOR=2.65, 95%, CI=1.12-6.30], birth attended by healthcare professional [AOR=3.20, 95% CI=1.23-8.31], source of information [AOR=3.89, 95%, CI=1.54-9.82] and good knowledge on colostrum feeding and breastfeeding [AOR=3.53, 95%, CI=1.56-7.96] were found to be associated with practice of colostrum feeding.

**Conclusion:**

in the present study, mothers who had good colostrum feeding practices were low. Socio-demographic, obstetrics, and knowledge-related factors were associated with colostrum feeding practice. Promoting antenatal care visits and maternal education on colostrum feeding is recommended to increase the colostrum feeding practice of the mothers.

## Introduction

Colostrum is the first milk or a sticky yellow fluid secreted by the mammary glands of mammals in late pregnancy just before giving birth and continuing through the early days of breastfeeding [1]. Colostrum is very rich in proteins, carbohydrates, vitamin A, sodium, chloride, various cytokines immunoglobulin, and growth factors, because of these combinations it encourages optimal development of the infant's heart, brain, and central nervous system and it also offers premature infants the best chance for the optimal development of their fragile organs [2-4].

Colostrum is considered as the baby's first immunization, and it has a positive effect on the prevention of childhood illness and malnutrition [5,6]. It also encourages the first passage of stool, which intern helps to clear excess bilirubin [4]. In addition, colostrum has advantages to the mother's health by increasing the postpartum infertility period, helping them return to their pre-gestational weight, and reducing their risk of breast and ovarian cancer [7]. Colostrum feeding (CF) has significant effects on the immediate and future health of newborn infants. It prevents preventable causes of neonatal morbidity like diarrhea and acute respiratory infection [8,9]. The WHO and UNICEF recommended exclusive breastfeeding (EBF) for children up to six months of age and it also recommended practicing timely initiation of breastfeeding, on-demand breastfeeding, and no use of bottles, teats, or pacifiers, and nourishing them with appropriate complementary foods and continued breastfeeding until 24 months or beyond [5,10]. Even though the WHO and UNICEF recommended initiating CF within the first hour of birth [10], still a higher number of mothers avoided their colostrum before giving milk to their infant for various societal myths and misconceptions [11]. Instead, they give honey, sugar water, glucose, and butter as a substitute; consequently, these man-made problems affect directly and indirectly the health of newborn infants and cause malnutrition and a high mortality rate in infants [12]. Avoiding colostrum in the first three crucial days after birth increases the risk of infection and death among neonates, and the risk of neonatal death was fourfold higher in children given milk-based fluids or solids in addition to breast milk [13].

Different studies show that the main contributing factor to stunting, wasting, and underweight among under-five children was a deprivation of CF [14-18]. Infants who were fed colostrum were less likely to be stunted and wasted compared to children who were deprived of colostrum [14,18,19]. Despite WHO and UNICEF recommendations, mothers in many parts of the country still discard colostrum because they considering it as heavy, dirty milk, harmful to children´s health, having no nutritional value, or see it as bad luck for the family and the infant [20-23]. The objectives of this study were to determine colostrum feeding practice (CFP) and to identify factors associated with CFP among mothers who attended a child immunization clinic in Dire Dawa City, Eastern Ethiopia.

## Methods

**Study design and period:** a health facility-based cross-sectional study design was employed from February 1/2021 to March 2/2021 at the public health facilities of Dire Dawa City.

**Study setting:** the study was conducted in Dire Dawa City health facilities. It is located about 515 km away from the capital city of Ethiopia, Addis Ababa, and 47 km from Harar Town. It has a hot temperature with a mean of 25 degrees centigrade. The city has ten public health facilities, among them, two of them are hospitals and eight of them are health centers. The city has a total population of 506,936, of these, 248,298 are male and 258,638 are female. It has 38 rural and 9 urban Keeble's (the lowest administrative unit in Ethiopia, next to the district) [24].

**Study participants:** all mothers who had babies less than or equal to twelve months and attended the child immunization clinic at the selected public health facilities of Dire Dawa City were included, while mothers who gave preterm birth were excluded.

**Dependent variable:** colostrum feeding practice.

### Independent variables

***Socio-demographic factors:*** age, residency, marital status, religion, educational level, occupation, partner educational status, and with whom she was living

***Reproductive and obstetrics factors:*** parity, ANC visit, counseling on timely initiation of breastfeeding, counseling on CF and breastfeeding during ANC visits, place of delivery, birth attendant, mode of delivery, counseling on breastfeeding immediately after delivery. Knowledge, practice, and source of information-related factors.

### Operational definitions

***Colostrum:*** it is the yellowish breast milk produced within the first few days after delivery [25].

***Colostrum feeding practice:*** the behaviour, habit, or custom of mothers of infants on CF to their current infants. The mother was considered to have good CFP if she correctly answered >60% of the total CFP assessing questions [26].

***Knowledge:*** refer to knowledge of the mothers about colostrum and breastfeeding. A total of 14 items, were used and for each item, those who responded “Yes” were scored (+1), and those who responded “No” scored (0). The mother was considered to have good knowledge if she correctly answered >60% of the total knowledge assessing questions [26].

**Sample size determination and techniques:** the sample size was calculated by using Epi info statistical software version 7.1 using two population proportion formula and considering giving childbirth with the assistance of a healthcare professional as a factor associated with CF from a study conducted in Wolaita Sodo City (AOR =3.12 and % of outcome in unexposed was 54.9% [25]. In this regard, a 5% level of significance (two-sided) or the hypothesis of no significant difference, a power of 80%, and the ratio of unexposed to exposed one was assumed. Based on the above assumption the sample size was 132 and after considering a 10% of non-response rate and design effects of 2, the final sample size was 292 mothers. A multistage sampling technique was done to select the study population. By lottery method, one hospital and three health centers were selected. The total sample size was proportionally allocated for each health facility of the administrative city based on their monthly expanded program of immunization unit flow. According to the expected number of women in the specified period of data collection, the sample size was proportionally allocated to those health facilities. Finally, the study participants were selected by using a systematic sampling technique. The starting unit was selected by using the lottery method among the first Kth units in each health facility.

**Data collection tools and procedures:** the questionnaire was developed by authors after reviewing different kinds of literature on the topic and validated by professional experts. Data were collected using structured and pre-tested interviewer-administered questionnaires through face-to-face exit interviews, which consist of socio-demographic characteristics, obstetric information, knowledge, and practice-related questions. The questionnaire was first prepared in English and then translated to Amharic and back to English again by a language expert to maintain consistency. Knowledge and CFP questions were assessed by '+1' for correct answers and '0' for an incorrect answer. The score for each mother was summed and categorized. The data were collected by four BSc midwives and nurses and supervised by one public health officer.

**Data quality control:** the data collectors and supervisors trained for two days by the investigator on the objectives, relevance of the study, ethical concerns, and techniques of interviews were given before the actual data collection. The questionnaire was pre-tested before the actual data collection period on 5% (15) mothers who attended the child immunization clinic in Sabian General Hospital, which is not selected in this study, then, the instrument was amended accordingly. The completeness of the data was checked by data collectors during data collection and daily supervision was done for data completeness. Data coding and entry were checked throughout the work. Data cleaning was checked at the end of the data entry.

**Data processing, analysis, interpretation, and presentation:** all the questionnaires were checked visually, and data cleaning, coding, and entry were done by using Epi data 4.2, then exported to SPSS version 25.0 for analysis. Descriptive and summary statistics like frequency, percentage, mean and standard deviation were carried out. Bivariate and multivariable logistic regression analyses were used to identify variables associated with CFP. During analysis, all explanatory variables that have a significant association in bivariate analysis with a P-value <0.20 were entered into a multivariable logistic regression model to get an adjusted odds ratio (AOR), and those variables with 95% of confidence intervals (CI) and a P-value of < 0.05 were considered as statistically significant with CFP. The multicollinearity between independent variables was checked by the variance inflation factor and was found acceptable (<2). The model goodness of the test was checked by using Hosmer-Lemeshow goodness of the fit and its P-value was 0.780. Frequency tables and descriptive summaries were used to describe the study variables.

**Ethical approval and consent to participate:** ethical clearance was obtained from the Institutional Review Board of Bahir Dar University, School of Chemical and Food Engineering Department of Applied Human Nutrition, and a letter of permission was obtained from the Dire Dawa City, Health Bureau. Written consent was obtained from each study participant. Confidentiality of information and privacy was maintained.

## Results

### Socio-demographic characteristics of the mothers

A total of 292 mothers participated in the study with a response rate of 100%. The mean age of the mothers was 24.72 years with (±SD=4.55) and 134 (45.9%) of the mothers were found in the age group of 20-25 years. Of the mothers, 235 (80.5%) lived in urban, and 278 (95.2%) were married. About 151 (51.7%) were followers of Muslim, and 115 (39.4%) had a primary education level. Of the mothers, 165 (56.5%) were housewives, and 87 (31.3%) of their partners had a secondary educational level (Table 1).

**Table 1:** socio-demographic characteristics of the mothers in Dire Dawa City, Eastern Ethiopia, 2021, (n=292)

Variables	Categories	Frequency	Percent
Maternal age in years	15-19	19	6.5
	20-25	134	45.9
	26-30	106	36.3
	>31	33	11.3
Residence	Rural	57	19.5
	Urban	235	80.5
Religion	Orthodox	129	44.2
	Muslim	151	51.7
	Others*	12	4.1
Marital status	Married	278	95.2
	Others **	14	4.8
Educational level	No formal education	47	16.1
	Primary education	115	39.4
	Secondary education	75	25.7
	Diploma and above	55	18.8
Occupational status	Housewife	165	56.5
	Merchants	78	26.7
	Employed (GO/NGO)	49	16.8
Partner educational level (n=278)	No formal education	35	12.6
	Primary education	80	28.8
	Secondary education	87	31.3
	Diploma and above	76	27.3
**Mother living**	with Partner	252	86.3
	With other else	40	13.7

* Catholic and Protestant, ** Single, Widowed and Divorced

### Obstetric characteristics of the mothers

In this study, 218 (74.7%) of the mothers were multigravida, and 238 (81.5%) had a history of the ANC visit in their most recent pregnancy. Among mothers who had a history of ANC visits, 195 (81.9%) were counseled on TIBF during their ANC visits. Of the mothers, 176 (73.9%) and 189 (79.4%) were counseled on CF and EBF during their ANC visit. About, 248 (84.9%) gave childbirth at a health facility, and 253 (86.6%) of the births were attended by health professionals. Of the mothers, 255 (87.3%) were given childbirth vaginally, and 232 (79.5%) of the mothers were counseled about breastfeeding immediately after delivery (Table 2).

**Table 2:** reproductive and obstetric characteristics of the mothers in Dire Dawa City, Eastern Ethiopia, 2021, (n=292)

Variables	Categories	Frequency	Percentage
Parity	Primipara	74	25.3
	Multipara	218	74.7
History of ANC visits	Yes	238	81.5
	No	54	18.5
Counseled on TIBF during your ANC visits (n=238)	Yes	195	81.9
	No	43	18.1
Counseled on CF during your ANC visits (n=238)	Yes	176	73.9
	No	62	26.01
Counseled on EBF during your ANC visits (n=238)	Yes	189	79.4
	No	49	20.6
Place of delivery	Home	44	15.1
	Health facility	248	84.9
Birth attendants	Health professional	253	86.6
	TBA/family	39	13.4
Mood of delivery	Vaginal delivery	255	87.3
	Caesarean section	37	12.7
Counseled on breastfeeding after delivery	No	60	20.5
	Yes	232	79.5

### Knowledge of colostrum and breastfeeding

Based on the predetermined criteria, 187 (64.0%) mothers had good knowledge of colostrum and breastfeeding. More than half (51.1%) of the mothers obtained their information on colostrum and breastfeeding from health professionals. Of the mothers, 263 (90.1%) responded that colostrum is the mother's breast milk during the first three days of delivery, and 161 (55.1%) of them knew that colostrum is nutritious and hygienic. Nearly 85% of the mothers responded that colostrum is the best first milk given to the baby, and 280 (95.9%) said that early initiation of breastfeeding with colostrum strengthens baby-mother bonding. Of the mothers, 170 (58.2%) of the mothers knew that early initiation of breastfeeding with colostrum prevents breast pain/engorgement, and more than two-thirds of the mothers (69.9%) responded that the baby should fed colostrum and breast milk on demand day, and night without provision of any PLF (Table 3).

**Table 3:** knowledge of colostrum and breastfeeding among mothers in Dire Dawa City, Eastern Ethiopia, 2021, (n=292)

Variables	Categories	Frequency	Percentage
Source of information (multiple responses are possible)	Health professional	149	51.1
	Mass media	69	23.6
	Family/Friends	76	26.1
Colostrum is the mother's breast milk during the first three days of delivery	Yes	263	90.1
	No	29	9.9
Color of colostrum	Yellow	288	98.6
	White	4	1.4
Breastfeeding started within an hour after delivery and continues for the first 3 days	Yes	212	72.6
	No	80	27.4
Colostrum is nutritious and hygienic?	Yes	161	55.1
	No	131	44.9
Colostrum is the best first milk given to the baby	Yes	248	84.9
	No	44	15.1
Early initiation of breastfeeding strengthens baby-mother bonding	Yes	280	95.9
	No	12	4.1
Early initiation of breastfeeding prevents breast pain/engorgement after birth.	Yes	170	58.2
	No	122	41.8
Early initiation of breastfeeding with colostrum within prevents vaginal bleeding after birth.	Yes	139	47.6
	No	153	52.4
The baby should breastfeed on demand day and night without the provision of any pre-lacteal feeding.	Yes	204	69.9
	No	88	30.1
Colostrum is important for the growth and development of the baby	Yes	154	52.7
	No	138	47.3
Colostrum gives natural immunity to the baby	Yes	109	37.3
	No	183	62.7
Child feed colostrum' when the mother is sick.	Yes	205	70.2
	No	87	29.8
Child feed colostrum’ when he/she is sick	Yes	233	79.8
	No	59	20.2
Colostrum protects the newborn from diseases	Yes	101	34.6
	No	191	65.4
Knowledge of colostrum and breastfeeding	Good knowledge	187	64.0
	Poor knowledge	105	36.0

### Colostrum feeding practice

In this study, according to the predetermined criteria, 201 (68.8%) mothers had good CFP. Of the mothers, 259 (88.7%) gave colostrum to their babies after birth, while 33 (11.3%) were not fed colostrum. Among mothers who didn´t provide colostrum, 13 (39.4%) mentioned it may cause abdominal discomfort and diarrhea for the baby as their main reason for not feeding colostrum. Of the mothers, 43 (14.7%) were given pre-lacteal feeding to their baby and among them, 16 (37.2%) gave infant formula milk, while 17 (39.5%) were mentioned cultural practice as their main reason for PLF. Of the mothers, 203 (69.5%) were starting colostrum feeding with breast milk within an hour of delivery, and 287 (98.3) were continuing to give colostrum with breast milk for the first three days of delivery (Table 4).

**Table 4:** colostrum feeding practice of the mothers in Dire Dawa Administrative City, Eastern Ethiopia, 2021, (n=292)

Variables	Categories	Frequency	Percentage
**Did you feed colostrum to the baby after birth**	Yes	259	88.7
	No	33	11.3
**Reason for not feeding colostrum (n=33)**	Causes abdominal discomfort	10	30.3
	Maternal illness	5	15.2
	Not clean	8	24.2
	My breast has no breast milk	6	18.2
	Baby unable to suck	4	12.1
**Pre-lacteal feeding**	Yes	43	14.7
	No	249	85.3
**Reason for pre-lacteal feeding (n=43)**	Cultural practice	17	39.5
	Not having enough milk	14	32.6
	Breast pain	7	16.3
	I was sick	5	11.6
**Types of pre-lacteal feeding (n=43)**	Infant formula milk	16	37.2
	Cow’s milk	11	25.6
	Plain water	7	16.3
	Sugar solution	5	11.6
	Honey	4	9.3
**How long after delivery did you put the baby on the breast**	Immediately within an hour	210	71.9
	After an hour	82	28.1
**The time mothers started breastfeeding**	Within an hour	203	69.5
	Within 6 hours after delivery	48	16.4
	Within 24 hours after delivery	12	4.1
	After discarding some of the colostrum	19	6.5
	After white milk appeared	10	3.4
**Did you feed the baby breast milk within the first three days after delivery**	Yes	287	287 98.3
	No	5	5 1.7
**Practice of CF**	Good practice	201	68.8
	Poor practice	91	31.2

### Factors associated with colostrum feeding practice

Bivariate analysis showed that maternal age, residency, educational level of the mothers, parity, history of ANC visit, counseling on TIBF, CF, and EBF, place of delivery, giving childbirth with the assistance of health care provider, counseling on breastfeeding after delivery, source of information and good knowledge of colostrum and breastfeeding were candidate variables for multivariable analysis at a P-value of less than 0.2.

In a multivariable analysis women who live in urban [AOR=2.52, 95% CI=1.08-5.88], mothers who had secondary and above educational level [AOR=2.79, 95% CI=1.12-6.98], having a history of ANC visit for the index pregnancy [AOR=3.12, 95% CI=1.12-9.21], mothers who are counseled on TIBF [AOR=2.59, 95%, CI=1.02-6.59], mothers who are counseled on CF [AOR=2.65, 95%, CI=1.12-6.30], giving childbirth with the assistant of health care professional [AOR=3.20, 95% CI=1.23-8.31], having information about colostrum and breastfeeding from healthcare professionals [AOR=3.89, 95%, CI=1.54-9.82] and mothers who had good knowledge of colostrum and breastfeeding [AOR=3.53, 95%, CI=1.56-7.96] were significantly associated with CFP at a P-value of less than 0.05 (Table 5).

**Table 5:** logistic regression analysis for colostrum feeding practice among mothers in Dire Dawa City, Eastern Ethiopia, 2021, (n=292)

Variables	CFP		COR (95%-CI)	AOR (95%-CI)	P-value
	Good	Poor			
**Maternal age in years**					
15-19	10	9	1	1	
20-25	95	39	2.19 (0.83-5.81)	1.77 (0.46-6.79)	0.403
26-30	73	33	1.99 (0.74-5.36)	2.87 (0.73-11.26)	0.131
>=31	23	10	2.07 (0.64.6.65)	2.11 (0.43-10.41)	0.357
**Residency**					
Rural	25	32	1	1	
Urban	176	59	3.82 (2.09-6.96)	**2.52 (1.08-5.88)**	0.033*
**Maternal educational level**					
No-formal education	21	26	1	1	
Primary education	79	36	2.72 (1.35-5.46)	2.36 (0.93-6.01)	0.072
Secondary and above	101	29	4.31 (2.12-8.75)	**2.79 (1.12-6.98)**	0.028*
**Parity**					
Primipara	48	36	1	1	
Multipara	153	55	2.09 (1.23-3.55)	1.30 (0.60-2.79)	0.506
**History of ANC visits**					
No	12	42	1	1	
Yes	189	49	13.50 (6.61-27.58)	**3.21 (1.12-9.21)**	0.030*
**Counseled on TIBF**					
No	38	60	1	1	
Yes	163	31	8.30 4.75-14.52)	**2.59 (1.02-6.59)**	0.046*
**Counseled on CF**					
No	52	64	1	1	
Yes	149	27	6.79 (3.92-11.77)	**2.65 (1.12-6.30)**	0.027*
**Counseled on EBF**					
No	49	53	1	1	
Yes	152	38	4.33 (2.55-7.33)	0.56 (0.21-1.50)	0.252
**Place of delivery**					
Home	22	22	1	1	
Health institution	179	69	2.59 (1.35-4.98)	0.68 (0.05-9.23)	0.772
**Birth attendant**					
TBA/family	19	20	1	1	
Health care professional	182	71	2.70 (1.36-5.35)	**3.20 (1.23-8.31)**	0.017*
**Counseled on breastfeeding immediately after delivery**					
No	29	31	1	1	
Yes	172	60	3.06 (1.71-5.50)	1.85 (0.59-5.76)	0.290
**Source of information**					
Family/friends	34	38	1	1	
Mass media	43	24	2.01 (1.01-3.96)	2.47 (0.99-6.15)	0.052
Health care professionals	124	29	4.78 (2.58-8.83)	**3.89 (1.54-9.82)**	0.004*
**Knowledge of colostrum and breastfeeding**					
Poor	49	56	1	1	
Good	152	35	4.96 (2.92-8.44)	**3.53 (1.56-7.96)**	0.002*

* Significant at a P-value of less than 0.05

## Discussion

Worldwide, more than 2.6 million neonates die each year, most of which occur within the first 7 days of birth, with about 1 million dying on the first day and close to 1 million dying within the next 6 days [10]. There are various factors that can effectively reduce neonatal mortality to greater levels, among those factors, colostrum feeding is recognized as the first and vital step toward reducing mortality in infants and children under five years of age and it also has the potential to prevent around 20% of newborn deaths and 13% of under-five deaths [9]. This study identified about 68.8% [95% CI: 63.4-74.3] of mothers who had good CFP. This result showed that CFP in the study area did not meet the WHO and UNICEF recommendation, which states that breast milk or colostrum should be the first taste of the newborn baby [5,10]. This finding was in line with previous studies conducted in different parts of Ethiopia: Harar Town Governmental Hospital 70.0% [27], Debre Tabor Referral Hospital 69% [28], rural pastoralist communities of Afar Region 63.6% [29], Boditi Town 72.5% [30], and Jimma Arjo Woreda 72.5% [22]. It was also consistent with studies conducted in rural Papua New Guinea 68.6% [31], Nepal 69% [32], Pakistan 72.1% [33], and Bangladesh 63% [34]. However, it was lower than studies conducted in different parts of Ethiopia: Debre Markos Town 77.51% [35], Bahir Dar City 83.3% [36], Arba Minch Zuria 89.0% [37], Wolaita Sodo City 87.4% [25], Motta Town 79.8% [38], Jinka Town 90.2% [39], East Wollega Zone 91.2% [40], Dembecha District 76.2% [41], Gozamen District 77.9% [42], Raya Kobo District 86.5% [23], Mekelle health facilities 80.5% [43], Kombolcha Town 88.6% [44], Axum Town 93.7% [45], North Wollo Zone 88.0% [46], North Eastern Ethiopia 88.9% [47], and Bure District 85.5% [48]. The possible reason for this inconsistency of CFP might be due to cultural differences of the study participants as Ethiopia population has different multi-cultural practices.

It was also lower than studies conducted in different countries: Teaching Hospital in Nepal 80% [32], Kamrup, India 79.0% [49], Bangladesh 75.92% [50], and Burkina Faso 84% [51]. The difference between these studies might be due to the difference in cultures, community attitudes towards CF, and socio-demographic characteristics of the study population. On the contrary, the findings in this study were higher than those studies conducted in Mizan Tepi University Teaching Hospital 60.9% [52], and Ambo District 56.5% [26]. The possible reasons for the better CFP in our study might be due to the time gap of the year of the studies. The other possible reason might be due to the increasing utilization of maternal health services over time, this may increase the chance of getting information related to CF in the form of health education or counseling. Similarly, it was also higher than the studies conducted in India 8.0% [53], South Sudan 61.2% [54], and Egypt 41.4% [55]. The difference may be attributed to the studies sitting and the difference in the cultural practice of the study population. Factors that positively affect the CFP were women's socio-demographics, obstetric characteristics, and knowledge of breast and colostrum feeding. Living in an urban aera increased the odds of having a good CFP by 2.52 times. This finding was supported by studies done in the Amibara District, and Teaching Hospital in Nepal [32,56]. As well as studies conducted in Debre Tabor General Hospital, Kombolcha, and Kamrup Assam India town show that mothers who lived in urban were more likely to feed colostrum than those who lived in rural [44,49,57]. The possible reason for this difference in CFP among urban and rural mothers might be due to the educational levels, as most of the mothers who live in urban are educated and have better knowledge of colostrum. The other possible reason might be due to cultural influences, mothers who live in rural areas have various societal myths and misconceptions.

Mothers who had secondary and above educational levels were 2.79 times more likely to have good CFP. The possible reason might be mothers who are educated could have better information regarding the advantages of CF. This finding was in line with a study done in the Amibara District [56]. It was also supported by studies conducted in Debre Tabor General Hospital, and Jimma Arjo Woreda shows that mothers with non-formal educational status, are more likely to discard colostrum than those who have formal education [22,57]. Moreover, a study conducted on urban and rural mothers in Kamrup Assam, India shows that having a low educational level is associated with colostrum avoidance [49]. On the other hand, mothers who had a history of the ANC visit for their index pregnancy were 3.21 times more likely to have good CFP. This finding agrees with another study done in Ethiopia [58]. This might be because having ANC visits could offer a good educational channel regarding neonatal feeding and the importance of CF to the neonate. Similarly, mothers who are counseled on TIBF were 2.59 times more likely to have good CFP. This finding was in line with another study [25]. This shows that lack of counseling on the importance of early initiation of breastfeeding makes the mothers initiate breastfeeding later, they would have more time for infant feeding malpractices like colostrum avoidance. This is supported by studies done in the Gozamen District and Kombolcha showing that mothers who did not get counseling on timely initiation of breastfeeding were more likely to discard colostrum than those counseled [42,44]. Additionally, studies conducted in Jinka Town, North Wollo Zone, Raya Kobo District, Nepal, Pakistan, and Uttarakhand India show that delayed initiation of breastfeeding is associated with colostrum avoidance [23,32,33,39,46,53].

Mothers who are counseled on CF were 2.65 times more likely to have good CFP. This finding was in line with other studies [40,59,60]. A study conducted in Ambo shows that a lack of information on CF was more negatively associated with CFP than those who have information on CF [26]. As well as studies conducted in Jinka and Debre Tabor General Hospital show that a lack of counseling about breastfeeding during ANC visits increases the chance of colostrum discarding [39,57]. This might be due to that counseling during ANC visits is the tool to change the behaviors of mothers to reduce postnatal nutritional malpractice such as pre-lacteal feeding and colostrum avoidance. ANC visits are the ideal time to increase a mother's knowledge of colostrum and breastfeeding. However, mothers who received no counseling about CF might have a low level of awareness of the benefit of CF for newborns. As a result, they may discard colostrum immediately after delivery. There is a supporting report from a study conducted in Ethiopia that shows that mothers who did not receive breastfeeding counseling during ANC visits were 73.9% more likely to avoid colostrum than their counterparts [58].

Giving childbirth with the assistance of a healthcare professional increased the odds of having a good CFP by 3.20 times. This finding was supported by other studies ^[25,29,38]^. The possible explanation for this might be that giving birth at the health institution with the assistance of health professionals could increase the chance of getting counseling on early initiation of breastfeeding relative to those who gave birth at home with the assistance of traditional birth attendants or family. There is a supporting report from a study conducted in Ambo that shows that giving childbirth at home was more negatively associated with CFP than those who gave birth at health institutions with the assistance of a health professional [26]. As well as a study conducted in the Bure district shows that mothers who gave birth at home were more likely to discard colostrum relative to those who gave birth at a health institution [48]. Mothers who got information about colostrum and breastfeeding from health professionals were nearly four times more likely to have good CFP. In this study, more than half (51.1%) of the mothers received information on colostrum and breastfeeding from health professionals. This showed that the information on colostrum obtained from a health professional is more accurate and the mothers have a good level of information on CF than those who obtained the information from other sources. There is a supporting study conducted in Aksum town shows that poor maternal level of information on CF more likely associated with the practice of colostrum avoidance than CF [45]. Additionally, mothers who had good knowledge of colostrum and breastfeeding were 3.53 times more likely to have good CFP. This finding was in line with studies conducted in Uttarakhand India, Raya Kobo, Axum Town, Hula District of Sidama Region, Jinka Town, and Bure District showing that mothers who had poor knowledge of breastfeeding were more likely to discard colostrum relative to those who had good knowledge of breastfeeding practice ^[23,39,45,48,53,60]^. This supporting evidence revealed that improving the mother's knowledge of colostrum and breastfeeding increases the likelihood of having a good CFP.

**Limitation:** this study was not triangulated with the qualitative method

## Conclusion

Despite the WHO and UNICEF recommendations, the CFP among mothers who have an infant less than twelve months of age was low in the study area. Among the predictors: residency, educational level, having a history of ANC visits, counseled on TIBF and CF during ANC visits, giving childbirth with the assistance of healthcare professionals, getting information about colostrum and breastfeeding from a health professional, and having good knowledge of colostrum and breastfeeding was significantly associated with CFP. Therefore, massive awareness creation on the CFP and avoidance of malpractices concerning CF, especially in the rural areas is needed. As well as promoting ANC visits for all pregnant women and maternal education on breastfeeding with colostrum is recommended for the increasing CFP of the mothers. Additionally, we also recommend designing and distributing booklets that highlight the importance of colostrum feeding.

### What is known about this topic

- Colostrum is very rich in proteins, carbohydrates, vitamins, and minerals and it also contains immunoglobulin;
- Infants fed colostrum were less likely to be stunted and wasted.

### What this study adds

- We found that getting information about colostrum and breastfeeding from health professionals could increase CFP;
- Having good knowledge of colostrum and breastfeeding has a direct relationship with CFP.
